# Urothelium-Specific Deletion of Connexin43 in the Mouse Urinary Bladder Alters Distension-Induced ATP Release and Voiding Behavior

**DOI:** 10.3390/ijms22041594

**Published:** 2021-02-05

**Authors:** Jin Kono, Masakatsu Ueda, Atsushi Sengiku, Sylvia O. Suadicani, Osamu Ogawa, Hiromitsu Negoro

**Affiliations:** 1 Department of Urology, Graduate School of Medicine, Kyoto University, Kyoto 606-8507, Japan; konojin@kuhp.kyoto-u.ac.jp (J.K.); adeu@kuhp.kyoto-u.ac.jp (M.U.); sengiku@kuhp.kyoto-u.ac.jp (A.S.); ogawao@kuhp.kyoto-u.ac.jp (O.O.); 2 Department of Urology, Tenri Hospital, Nara 632-8552, Japan; 3 Sengiku Urology Clinic, Shiga 524-0045, Japan; 4 Department of Urology, Albert Einstein College of Medicine, Bronx, NY 10461, USA; sylvia.suadicani@einsteinmed.org; 5 Department of Urology, University of Tsukuba, Ibaraki 305-8575, Japan

**Keywords:** urothelium, connexin43, gap junction, hemichannel, ATP

## Abstract

Connexin43 (Cx43), the main gap junction and hemichannel forming protein in the urinary bladder, participates in the regulation of bladder motor and sensory functions and has been reported as an important modulator of day–night variations in functional bladder capacity. However, because Cx43 is expressed throughout the bladder, the actual role played by the detrusor and the urothelial Cx43 is still unknown. For this purpose, we generated urothelium-specific Cx43 knockout (uCx43KO) mice using Cre-LoxP system. We evaluated the day–night micturition pattern and the urothelial Cx43 hemichannel function of the uCx43KO mice by measuring luminal ATP release after bladder distention. In wild-type (WT) mice, distention-induced ATP release was elevated, and functional bladder capacity was decreased in the animals’ active phase (nighttime) when Cx43 expression was also high compared to levels measured in the sleep phase (daytime). These day–night differences in urothelial ATP release and functional bladder capacity were attenuated in uCx43KO mice that, in the active phase, displayed lower ATP release and higher functional bladder capacity than WT mice. These findings indicate that urothelial Cx43 mediated ATP signaling and coordination of urothelial activity are essential for proper perception and regulation of responses to bladder distension in the animals’ awake, active phase.

## 1. Introduction

Gap junction channels and hemichannels play an essential role in the maintenance of cell homeostasis and the coordination of cellular activity in various organ systems [1]. Gap junctions, which are intercellular channels formed by the pairing and the docking of hemichannels from adjacent cells, provide a direct cytosol-to-cytosol pathway for exchange of ions and small molecules that couple the cells both electrically and metabolically. Unpaired hemichannels can also function as cell surface channels. This has been shown to occur with hemichannels formed by some of the connexins, including connexin43 (Cx43), which, in a such role, can participate in mechanisms of autocrine and paracrine signaling. In the urinary bladder, a growing body of evidence indicates that not only the Cx43 formed gap junctions [1,2,3,4,5,6,7] but also the Cx43 hemichannels have a significant role in micturition physiology [8]. Earlier studies of Cx43 in the bladder focused primarily on its role in detrusor function. Findings of increased Cx43 expression in the bladder were associated with bladder overactivity through increased gap junction formation and intercellular coupling [9]. However, the knowledge that Cx43 is expressed in all layers of the bladder [4,10], especially in the urothelium [8,11,12]—that is now also viewed for its role in regulating bladder function—and findings that Cx43 hemichannels can form functional channels in various cell types led to more recent studies aimed at further elucidating the role of Cx43 in bladder function.

The urothelium exerts both protective and sensory functions in the bladder. Protection of the bladder against the noxious urine contents is granted by the tight barrier formed by the highly specialized umbrella cells on the apical surface of the urothelium. The sensory function is granted by the expression of numerous receptors and channels and the ability of urothelial cell to respond to different stimuli with release of signaling molecules [13,14]. Of these, ATP is regarded as being the main transmitter released from the urothelium in response to bladder distension and urothelial ATP signaling as playing a key role in the activation of the bladder afferents that ultimately convey information to the CNS regarding the degree of bladder fullness. A few receptors and channels have been implicated in mechanisms of mechanosensitive urothelial ATP release. We recently demonstrated that these also include Cx43 hemichannels, which brings another participant in mechanisms of urothelial mechanosensory transduction that has to be considered for its potential role in modulating bladder function [8].

Bladder function follows a diurnal rhythm with a marked increase in capacity in the sleep phase and a decreased capacity in the active phase [15]. It has been revealed that this diurnal change of the bladder function is generated, at least in part, by a circadian change of Cx43 expression in both the detrusor smooth muscle cells and the urothelial cells [8,15,16]. However, little is known of the extent to which urothelial Cx43 actually contributes to bladder function, since prior in vivo studies that assessed changes in micturition behavior were conducted with global heterozygous Cx43 knockout mice, which could not distinguish the role of Cx43 in bladder smooth muscle, interstitial cells, and urothelium and could not omit the potential influence of altered Cx43 function in other tissues and organs [16,17].

In this study, to overcome these constraints and specifically evaluate the role of urothelial Cx43 on bladder function, we used the Cre recombinase-LoxP (Cre-LoxP) system and the *Uroplakin II (Upk2) Cre* mice to generate urothelium-specific Cx43 knockout (uCx43KO) mice. Here, we demonstrate that urothelium-specific Cx43 downregulation blunts the day–night differences in the urothelial ATP release response to bladder distension and disrupts the diurnal regulation of bladder capacity in the awake, active phase of the uCx43KO mice. The relevance of these findings is discussed in the overall context of mechanisms and molecular mediators of bladder function, and we highlight the significance and the potential role of urothelial Cx43 in bladder physiology.

## 2. Results

### 2.1. Urothelium-Specific Deletion of Connexin43

**Figure 1:**
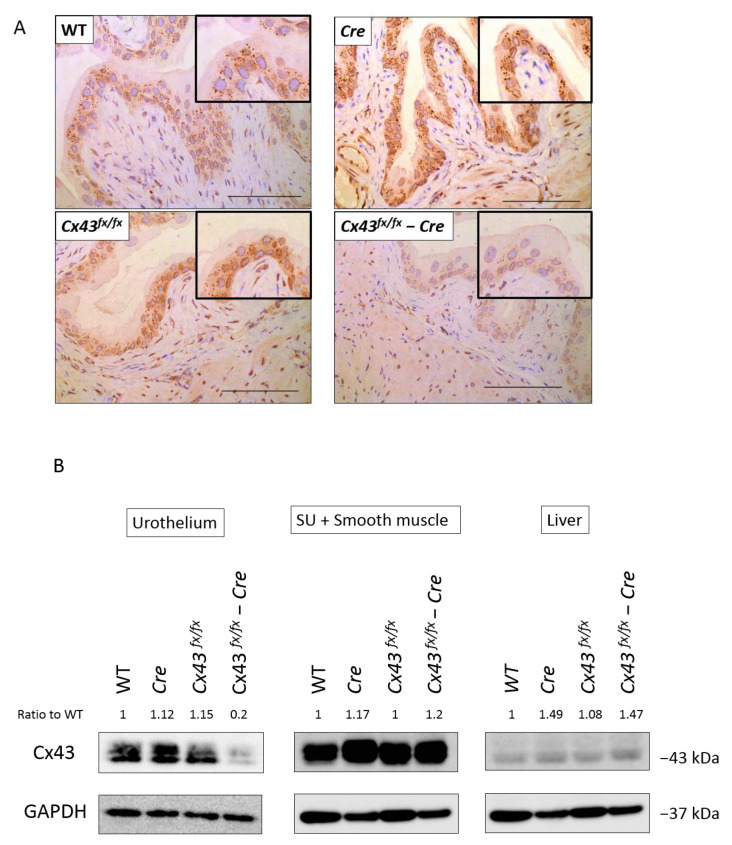
The *Upk2Cre+;* Connexin43 *Cx43^fx/fx^* mice (uCx43KO) are urothelium-specific connexin43 (Cx43) knockouts. (**A**) Immunohistochemistry of the bladder mucosa from wildtype (WT), *Upk2Cre+* (*Cre*), Floxed *Cx43* (*Cx43^fx/fx^*), and uCx43KO (*Cx43^fx/fx^*−Cre) mice. All scale bars indicate 100 µm. (**B**) Representative Western blots for Cx43 expression in bladder urothelium, suburothelium, and smooth muscle, and liver of from wildtype (WT), *Upk2Cre+* (*Cre*), Floxed *Cx43* (*Cx43 ^fx/fx^*), and uCx43KO (*Cx43^fx/fx^*−*Cre*) mice. “Ratio to WT” values presented above each lane correspond to Cx43/GAPDH normalized by WT Cx43/GAPDH values determined from the densitometric analysis of protein bands using the ImageJ software.

Floxed *Cx43* (*Cx43^fx/fx^*) mice were crossed with *Upk2Cre+* mice, and *Upk2Cre+; Cx43^fx/fx^* (denoted as “urothelium-specific Cx43” knockout; uCx43KO) were generated as well as *Cx43^fx/fx^* and *Upk2Cre+* mice. To confirm the deletion of Cx43 protein in the urothelium, we performed immunohistochemistry in the bladder of uCx43KO and control mice (*Cx43^fx/fx^, Upk2Cre+* and WT mice). In contrast to what was observed in the urothelium of control mice, the intensity of Cx43 staining in the urothelium of uCx43KO mice was clearly reduced, especially in the basal and the intermediate layers (Figure 1A). To further evaluate the specificity of urothelial Cx43 deletion in uCx43KO mice, immunoblotting analysis was performed to compare Cx43 protein expression in all the bladder layers and in the liver. The level of Cx43 expression was decreased in the urothelium of the uCx43KO mice, whereas in the suburothelium/detrusor muscle and in the liver, it was similar to those detected in control mice (Figure 1B). These findings confirm that the uCx43KO mice can be used as a urothelium-specific Cx43 knockout model.

### 2.2. Evaluation of Mechanosensitive Urothelial ATP Release in uCx43KO Mice

**Figure 2:**
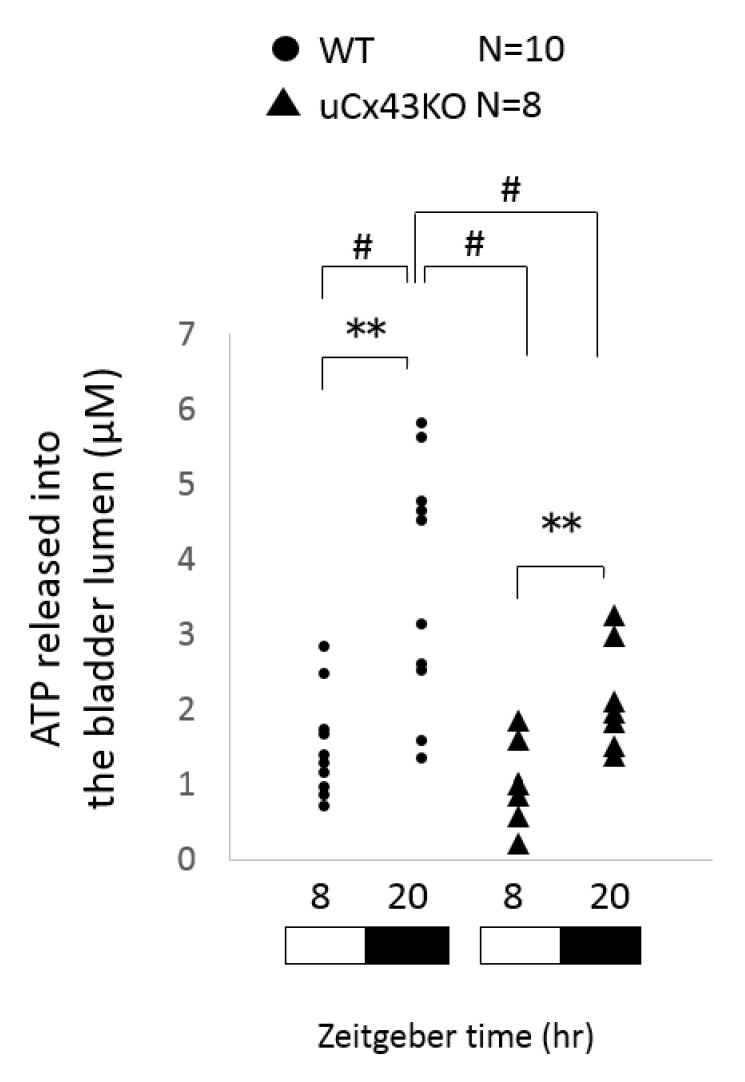
Comparison of the temporal changes in distension-induced luminal ATP release in WT and uCx43KO mice. Luminal ATP released amounts in response to bladder distension quantified from WT (*n* = 10) and uCx43KO (*n* = 8) mice during sleep phase (light phase; ZT8) and active phase (dark phase; ZT20). Note higher ATP release amounts from WT mice during the awake phase when compared to the sleep phase. This temporal difference remains in uCx43KO but is significantly attenuated when compared to WT mice. ZT, zeitgeber time: light on at ZT0 and off at ZT12. ** *p* < 0.01 Student’s *t*-test. # *p* < 0.01 versus the WT ZT20 group, one-way ANOVA followed Dunnett’s post hoc test.

Previously, we reported that Cx43 hemichannels participated in mechanisms of urothelial ATP release in response to bladder distension [8]. We also demonstrated that luminal ATP released levels underwent daily variations in mice that correlated with the diurnal expression of urothelial Cx43, and that these variations were diminished in the global *Bmal1*-knockout mice [8]. However, it is still unclear whether and to what extent the urothelial Cx43 expression influences the diurnal changes of the urothelial ATP release response to bladder distension. Hence, we compared this response in WT and uCx43KO mice at two points, meaning at the animals’ active phase (night/dark phase, as mice are nocturnal) and sleep phase (light phase). The temporal changes in the distension-induced ATP release response, characterized by an increase in ATP released amounts in the active phase and a decrease in the sleep phase, were observed in both WT mice and uCx43KO mice (Figure 2). However, the absence of urothelial Cx43 expression significantly attenuated this day–night difference in distension-induced ATP release in the uCx43KO mice when compared to WT mice (Figure 2). Particularly during the active phase, the luminal ATP concentration in uCx43KO mice was reduced significantly when compared to that in WT mice. 

**Figure 3:**
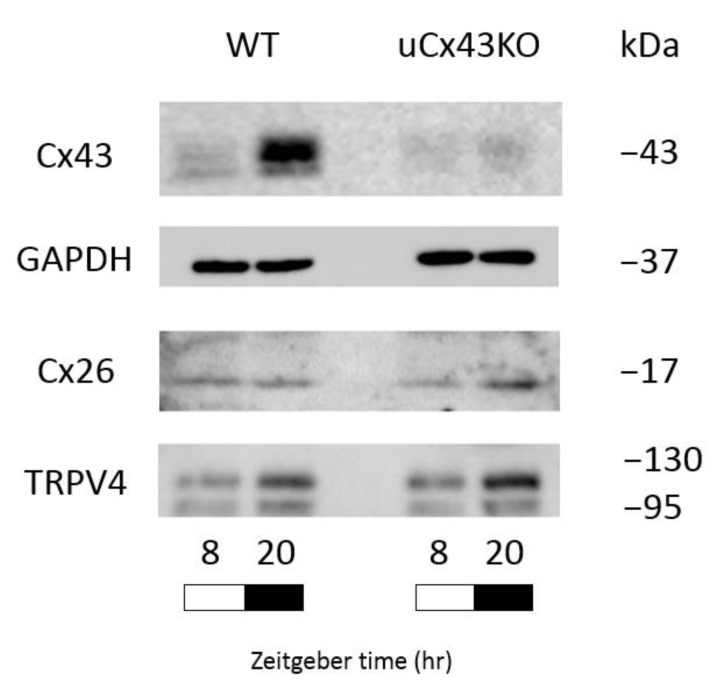
Urothelial protein expression of Cx43 and of other molecular mediators of urothelial ATP release. Representative immunoblots comparing Cx43 protein expression in the urothelium of WT and uCx43KO mice during sleep phase (light phase; ZT8) and active phase (dark phase; ZT20). Cx43 expression is barely detected in uCx43KO mice, while in WT mice, it displays a temporal expression pattern: low in the sleep and high in the awake phase. Other mediators of urothelial ATP release, such as connexin26 (Cx26) and transient receptor potential vanilloid 4 (TRPV4), are similarly expressed in the urothelium of WT and uCx43KO mice at both sleep and active phases. ZT, zeitgeber time: light on at ZT0 and off at ZT 12.

Previous studies by us and others [8,18] have shown that, in addition to Cx43, other molecular mediators of urothelial function, including transient receptor potential vanilloid 4 (TRPV4) and connexin26 (Cx26), have their expression following a similar diurnal oscillation and have also been implicated in mechanisms of urothelial ATP release. In this regard, to rule out the possibility of off-target effects of urothelial Cx43 deletion on TRPV4 and Cx26, we compared their expression in the urothelium of WT and uCx43KO mice. As shown in Figure 3, there were no significant differences in urothelial TRPV4 and Cx26 protein expression levels between WT and uCx43KO mice. These findings strongly indicate that Cx43 itself has a substantial role in mechanisms of mechanosensitive urothelial ATP release and suggest that its contribution to regulate the ATP release response to bladder distension is more apparent in the awake, active phase compared to the sleep phase of the mice.

### 2.3. Characterization of the Voiding Behavior of uCx43KO Mice

**Figure 4:**
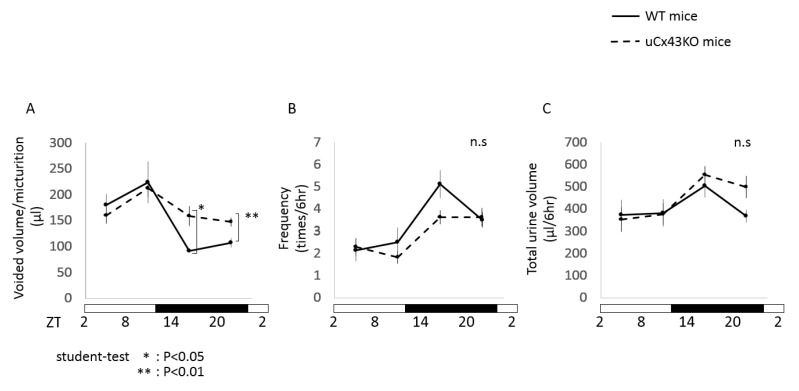
Comparison of micturition behavior of uCx43KO and WT mice assessed by the automated voided stain on paper (aVSOP) method. Temporal changes in average urine voided volume per micturition (**A**), voiding frequency (**B**), and total voided urine volume (**C**) of uCx43KO (*n* = 4) and WT (*n* = 4) mice determined from every 6 h along sleep (light) and awake (dark) phases. Note that WT and uCx43KO mice do not significantly differ regarding voiding frequency (**B**) and total voided volume (**C**) at both the sleep and the awake phases of the day. The voided volume per micturition (**A**) of WT and uCx43KO are also similar during the sleep phase, but during the awake phase, the voided volume per micturition is significantly higher in uCx43KO mice. ** *p* < 0.01 and * *p* < 0.05 by Student’s *t*-test. All error bars indicate s.e.m. ZT, zeitgeber time: light on at ZT0 and off at ZT 12.

To assess the effect of the urothelium-specific deletion of Cx43 on bladder function under physiological conditions, we compared the spontaneous voiding behavior of uCx43KO and WT mice using the automated voided stain on paper (aVSOP) method as previously described [16]. As shown in Figure 4A, a stark difference between uCx43KO and WT mice was observed during the animals’ active phase (dark phase) when the urine voided volume per micturition was significantly higher in uCx43KO mice than that in WT mice. The total urine volume as well as the voiding frequency of uCx43KO and WT mice were not significantly different. The observed difference in the voided volume per micturition between uCx43KO and WT mice inversely correlates with both the lower urothelial Cx43 expression in WT mice and the lower ATP concentration observed in both animals in the active phase. These findings indicate that urothelial Cx43 is required for proper regulation of the functional bladder capacity and generation of its diurnal rhythm, particularly in the awake, active phase.

## 3. Discussion

**Figure 5:**
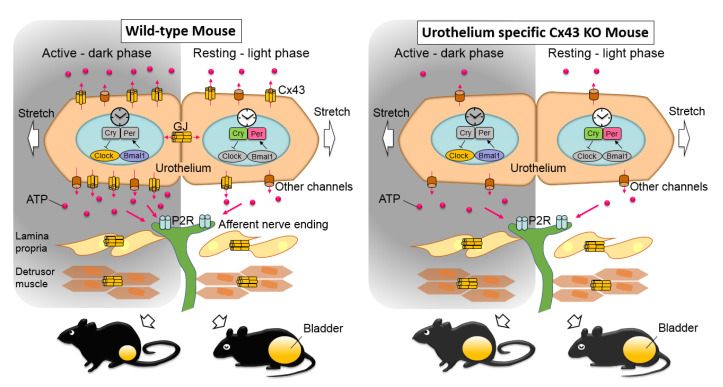
A schematic model of the events occurring in the wild-type mouse and in the urothelial specific Cx43 KO mouse, focusing on urothelial Cx43, ATP, and functional bladder capacity (Figure modified from supplement of [8]). In uCx43KO mice, downregulation of Cx43 expression in the urothelium results in reduced urothelial ATP release, especially in the animals’ active phase (night-time). The day–night differences in the urothelial ATP release and the functional bladder capacity are attenuated in uCx43KO mice that, in the active phase, displayed lower ATP release and higher functional bladder capacity than WT mice. Urothelial ATP signaling via Cx43 gap junction and hemichannel lead to stimulate P2 purinergic receptors (P2R) on the afferent nerve ending to modulate functional bladder capacity through other mechanisms. GJ, gap junction.

A convincing body of evidence has been accumulated that supports important roles for Cx43 in the bladder, including in mechanisms that regulate the functional bladder capacity [1,2,3,4,5,6,7,9,11,12,15,16,17,19]. Cx43 functionally connects the cells in the urothelial, the suburothelial, and the detrusor layers by forming gap junctions [6,9,16]. By forming hemichannels, Cx43 also participates in mechanisms of mechanosensitive urothelial ATP release, regulation of purinergic signaling, and bladder mechanotransduction [8]. However, because Cx43 is expressed throughout the bladder, it is unclear the extent to which urothelial Cx43 itself influences bladder function. In this study, we generated uCx43KO mice to clarify and distinguish the role of Cx43 expressed in the urothelium. The uCx43KO mice display urothelium-specific Cx43 protein suppression and no obvious anatomical abnormalities. Consistent with the proposed role of Cx43 hemichannels in urothelial ATP release and lack of protein expression, the amount of ATP released into the bladder lumen of uCx43KO mice after bladder distention tended to be lower when compared to control mice and was significantly decreased during the awake, active phase of the mice. The bladder function analysis showed a significant increase in the voided volume per micturition only in the active phase of the uCx43KO mice when compared to control mice, while no differences were observed in total urine voided volume or voiding frequency. These results suggested that suppression of the Cx43 gap junction-mediated coupling in the urothelium and Cx43 hemichannel contribution to urothelial ATP release blunts the coordination of urothelial responses to bladder distension, which attenuates the stimulation of urothelial cells and bladder sensory nerves, particularly during the awake, active phase of the animals, when the levels of Cx43 protein and of its contribution are the most important (Figure 5).

The sensory role of the urothelium and the importance of both urothelial ATP signaling and mechanisms of mechanosensitive urothelial ATP release for proper micturition function are well recognized. Earlier studies with P2X3 purinergic receptor KO mice demonstrated that absence of these receptors attenuated the activation of the bladder afferent nerve fibers in response to bladder distension and to instillation of P2X3 receptor agonists, and that animals displayed increased bladder capacity and impaired micturition reflex [20,21]. Several other studies with transgenic models have since emphasized the importance of ATP signaling for proper urothelial mechanosensory transduction and have identified the involvement of various receptors and channels in mechanisms of urothelial ATP release and evaluated the extent to which their absence impacted bladder function [13]. It is thus not surprising that purine receptors and other molecular components of the urothelial mechanosensory and transduction system are drawing growing attention for their potential as therapeutic targets for conditions involving altered voiding behavior or urinary perception. However, this field is not without controversy. Recent reports show that, under physiological conditions, there are no remarkable differences in the micturition reflex or the voiding behavior of P2X3 purinergic receptor KO mice when compared to WT mice [22]. Additionally, in contrast to prior studies with TRPV1 KO or TRPV4 KO mice [21,23,24] that showed reduced distension-induced urothelial ATP release and inhibition of micturition reflex induced by ATP instillation, recent studies have shown increased urinary frequency in TRPV1 or TRPV4 KO mice compared to WT mice [25]. This discrepancy may come from use of global knockout mice in these reports and differences in experimental approaches, since more physiological approaches that evaluate overall changes in voiding behavior can be influenced by changes in function of these molecular players on sites other than the urothelium, such as bladder detrusor, vessels, or central nerve system. In the present study, the use of urothelium-specific genetic manipulations allowed us to clearly determine the role played by urothelial Cx43 in mice under physiological conditions. To the best of our knowledge, such a urothelium-specific approach to investigate the mechanosensory role of the urothelium and ATP release in bladder function was used in only a few prior reports. In one report, the authors also used the Cre-LoxP system to generate urothelial-specific β1−integrin KO mice [26]. These mice displayed increased distension-induced ATP release that was accompanied by increased voiding frequency, suggesting a correlation between altered urothelial ATP signaling and bladder function. Another report disclosed the mechanosensory role of Piezo2 in both the sensory neurons and the bladder urothelium using the Cre-LoxP system [27].

Non-invasive, physiological assessments of mouse voiding behavior have shown that mice have a diurnal micturition rhythm with day–night changes in the voided volume per micturition, which we have shown in this study to be attenuated but still present in uCx43KO mice. The Cx43 formed hemichannels are not the only channels involved in mechanisms of urothelial ATP release, nor is ATP the only urothelial transmitter involved in the regulation of bladder volume. Other molecular mediators have also been associated with diurnal changes in ATP release, such as TRPV1, TRPV4, Piezo1 channels, and Cx26 hemichannels that would account for the remaining regulation of the day–night change in the ATP release and the voided volume per micturition observed in the absence of Cx43 [18]. Such involvement of more than one molecular player in urothelial mechanisms that regulate the functional bladder capacity denotes its physiological relevance and the high level of safety built to maintain it. Findings that we obtained in this study using the uCx43KO mice demonstrate that Cx43 plays a central role in these mechanisms, particularly in the animals’ awake, active phase. Initial evidence for the involvement of Cx43 in the regulation of functional bladder capacity was obtained from our earlier study with global *Cx43^+/−^* mice [16]. Interestingly, there are significant differences between the voiding behavior pattern of uCx43KO mice and *Cx43^+/−^* mice, as the later display higher voided volume per micturition not only during the active phase but also during the resting phase when compared to WT mice [16]. One interpretation for this discrepancy in the voided volume per micturition between the global *Cx43^+/−^* mice and the uCx43KO mice would take into account the effect that reduction on Cx43 gap junction coupling within the bladder would have on the level of activation and coordination of detrusor function, which can lead to a relatively large bladder volume. However, caution is also required with the interpretation of findings from *Cx43^+/−^* mice, as Cx43 is expressed in most tissues and organs, especially in brains, hearts, muscles, and endocrine systems [28,29], in which the reduction in Cx43 might have also influenced the changes in functional bladder volume aside from the effects imposed per se by decreased Cx43 expression in the bladder.

We recognize that this study has a few limitations. Firstly, the analysis of Cx43 function in urothelium focused on its role in forming hemichannels that participate in mechanisms of mechanosensitive urothelial ATP release and signaling. The Cx43 function in terms of its role in forming gap junctions and providing for coordination of urothelial responses to bladder distension was inferred but not experimentally demonstrated. Further studies are expected that will assess changes in junctional coupling and gap junction-mediated signaling in the urothelium of uCx43KO mice. Secondly, we refer to the uCx43KO mice as functional knockout, but in these mice, the expression of Cx43 in the urinary epithelium was not completely suppressed, and the very low but still detectable levels may be related to the distribution of Upk2 in the urothelium or the level of Cre recombinase expression. Thirdly, we examined the urothelial Cx26 and TRPV4 expression levels in uCx43KO mice, but other potential off-target effects or compensation for Cx43 deletion are yet unknown and cannot be discarded. Fourthly, uCx43KO mice are conditional knockout mice generated using a conventional Cre-LoxP system rather than using an inducible Cre-ERT2 recombinase technology. We attempted the generation of conditional urothelium-specific Cx43 KO mice by crossing *Upk2-CreERT2* mice [30] with *Cx43^fx/fx^* mice but obtained an unexpected Cx43 overexpression in the urothelium of the mice after tamoxifen induction. Hence, *Upk2Cre+* mice were used instead of *Upk2-CreERT2* mice for mating with *Cx43^fx/fx^* mice. In this regard, potential effect of Cx43 deletion on the urothelium during uCx43KO development cannot be ruled out, although no changes in urothelial structure were apparent from histological inspection (Figure 1). Bearing these limitations in mind, the findings presented in this study clearly showed the effect that specific deletion of Cx43 expression in the urothelium imposes on the diurnal changes in mechanosensitive urothelial ATP release and on the regulation of the voided volume per micturition.

In conclusion, Cx43 plays an important role in urothelial function, where it participates in mechanisms of urothelial ATP release in response to bladder distention, especially in the active phase when Cx43 expression is higher, which may modulate the diurnal change in the bladder capacity. Intercellular coupling provided by urothelial Cx43 gap junctions is also expected to support the transmission of signals within the urothelial and coordinate the responses to bladder distension, mainly in the active phase. Further studies are now needed to clarify the relationships between the urothelial Cx43 and other receptors and channels, the involvement of other proteins related to signal transmission in the urothelium, and the specific roles of Cx43 in the suburothelium and in the detrusor.

## 4. Materials and Methods

### 4.1. Animals

Eight-week-old female C57BL/6 mice were purchased from CLEA Japan (Tokyo, Japan). We obtained the *Upk2Cre+* mice and the *Cx43^fx/fx^* mice from the Jackson Laboratory (Bar Harbor, ME, USA). Bladder urothelium-specific Cx43KO mice were generated by crossing *Upk2Cre+* and *Cx43^fx/fx^* mice. Mice were housed at a constant room temperature with a cycle of 12 h light (7:00 to 19:00) and 12 h dark (19:00 to 7:00). Food and water were available ad libitum. This study was approved by the Kyoto University Animal Studies Committee (Permit Number: Medkyo19242) and complied with the guidelines for animal experimentation of the experimental animal center of the Kyoto University.

### 4.2. Immunohistochemistry of Mouse Bladder

Paraformaldehyde-fixed, paraffin-embedded bladders from control and uCx43KO mice were treated with citrate buffer for antigen retrieval, and immunohistochemistry was performed by the avidin–biotin complex (ABC) method. Anti-Cx43 (C6219, Sigma-Aldrich, St. Louis, MO, USA, 1:500) was used as the primary antibody against Cx43, and the biotinylated secondary antibodies (1:300) and the ABC kit were used following the manufacturer’s instructions (ABC-Elite, Vector Laboratories, Burlingame, CA, USA).

### 4.3. Tissue Harvesting

Eight-week-old female C57BL/6 mice and age-match female transgenic mice were sacrificed at ZT8 and ZT20 during the day under a dim light. Bladder tissues were then harvested and processed for biochemical analysis as previously described [8]. Briefly, the urothelium was gently scraped with a scalpel in cold normal saline then transferred with the normal saline to a 2 mL Eppendorf tube and spun down. The pellet was immediately cryopreserved in liquid nitrogen for protein assay. The remaining bladder tissue (suburothelium still attached to the detrusor smooth muscle) and a part of the liver were used as controls.

### 4.4. Immunoblotting

Bladder urothelium, suburothelium and smooth muscle layers, and liver tissue were lysed with radioimmunoprecipitation assay (RIPA) buffer containing protease inhibitors. Total cellular protein concentrations were determined using the detergent compatible (DC) protein assay reagent (Bio-Rad Laboratories, Richmond, CA, USA). Protein lysates (20 μg) were subjected to SDS-PAGE using 10% gel and transferred to polyvinylidene difluoride membranes (Millipore, Bedford, MA, USA) using a Mini Trans-Blot Cell system (Bio-Rad Laboratories). Membranes were blocked with 1% bovine serum albumin (BSA) diluted in Tris-buffered saline with 0.1% Tween^®^ 20 detergent (TBST) and incubated with primary antibodies diluted in 1% BSA/TBST followed by incubation with horseradish peroxidase-conjugated secondary antibodies diluted in 1% BSA/TBST and bands detected by enhanced chemiluminescence with SuperSignal West Pico Chemiluminescent Substrate (Thermo Fisher Scientific, Waltham, MA, USA). Images were acquired with the LAS-4000 imaging system (Fujifilm Life Science, Tokyo, Japan). Anti-Cx43 (C6219, Sigma-Aldrich, St. Louis, MO, USA, 1:10000), Anti-Cx26 (Life Technologies/Invitrogen, Waltham, MA, USA, 1:500), anti-TRPV4 (ab39260, Abcam, Cambridge, UK, 2 µg/mL), and anti-GAPDH (2118, Cell Signaling Technology, Danvers, MA, USA, 1:5000) were used as primary antibodies. Levels of Cx43 and GAPDH (loading control) protein expression were quantified using the ImageJ software (National Institute of Health, Rockville Pike, MD, USA, http://rsb.info.nih.gov/ij/). Values were first normalized to respective loading control and are expressed relative to wildtype levels.

### 4.5. Bladder Distension and Quantification of Luminal ATP Release

Distension-induced urothelial ATP release into the bladder lumen was quantified from eight-week-old female C57BL/6 mice and from age-match female uCx43KO mice at both ZT8 (sleep/light phase) and ZT20 (active/dark phase). The experimental procedures were as previously reported [8]. Briefly, with animals under 2.0% isoflurane anesthesia, a 24 G catheter (SR-OT2419C, Terumo, Tokyo, Japan) was inserted into the urethra and held in place with a clamp (AM-1, Natsume Seisakusho Co. Ltd., Tokyo, Japan). The catheter was then connected to a tube filled with phosphate buffered saline (PBS) and a pressure reservoir. After bladder distention with 30 cm H_2_O for 10 min, the PBS from the bladder lumen was collected and snap-frozen in liquid nitrogen. The luciferin-luciferase assay (CellTiter-Glo Luminescent Cell Viability Assay; Promega, Madison, WI, USA) was used to quantify the amounts of ATP released. Briefly, 20 μL of the collected PBS samples were individually placed in triplicate in white walled 96-well plates (Nunc F96 MicroWell; Thermo Fisher Scientific, Waltham, MA, USA), and 20 μL CellTiter-Glo^®^ 2.0 reagent was added directly to each well. Plates were incubated at room temperature for 10 min and then transferred to the Multilabel Plate Reader VICTOR X5 (PerkinElmer, Waltham, MA, USA), where luminescence was measured using a 1 s integration time.

### 4.6. Micturition Analysis

For continuous assessment of the voiding behavior of free-moving mice, the automated voided stain on paper (aVSOP) method was used as previously described [16]. Briefly, animals were kept in the aVSOP chamber in a sound-proof room and provided with free access to food and water. After adapting to the environment for 2 days, a laminated filtered paper was placed under the aVSOP chamber, and the micturition behavior was continuously assessed over 4 days.

### 4.7. Statistical Analysis

All data are expressed as the mean ± s.e.m. BellCurve for Excel (Social Survey Research Information Co., Ltd., Tokyo, Japan) was used for statistical analysis. Unpaired *t*-test and one-way ANOVA with Dunnett’s post hoc test were performed when appropriate. *p* < 0.05 was regarded as statistically significant. In the figures, statistical significance is indicated as follows: * *p* < 0.05 and ** *p* < 0.01.

## Data Availability

Not applicable.
